# Kinetic Release Studies of Antibiotic Patches for Local Transdermal Delivery

**DOI:** 10.3390/pharmaceutics13050613

**Published:** 2021-04-23

**Authors:** Esra Altun, Esra Yuca, Nazmi Ekren, Deepak M. Kalaskar, Denisa Ficai, Georgiana Dolete, Anton Ficai, Oguzhan Gunduz

**Affiliations:** 1 Centre for Nanotechnology & Biomaterials Research, Department of Metallurgical and Materials Engineering, Faculty of Technology, Goztepe Campus, Marmara University, Istanbul 34722, Turkey; esra.altun@ymail.com; 2 Department of Molecular Biology and Genetics, Faculty of Arts and Sciences, Davutpasa Campus, Yildiz Technical University, Istanbul 34220, Turkey; eyuca@yildiz.edu.tr; 3 Centre for Nanotechnology & Biomaterials Research, Department of Electrical-Electronics Engineering, Faculty of Technology, Goztepe Campus, Marmara University, Istanbul 34722, Turkey; nazmiekren@marmara.edu.tr; 4 UCL Division of Surgery and Interventional Science, Royal Free Hospital Campus, University College London, Rowland Hill Street, London NW3 2PF, UK; 5 Department of Inorganic Chemistry, Physical Chemistry and Electrochemistry, Faculty of Applied Chemistry and Materials Science, University POLITEHNICA of Bucharest, 060042 Bucharest, Romania; denisaficai@yahoo.ro; 6 National Centre for Micro- and Nanomaterials, University POLITEHNICA of Bucharest, 060042 Bucharest, Romania; dolete.georgiana@gmail.com; 7 Department of Science and Engineering of Oxide Materials and Nanomaterials, Faculty of Applied Chemistry and Materials Science, University POLITEHNICA of Bucharest, 060042 Bucharest, Romania; 8 Academy of Romanian Scientists, 060042 Bucharest, Romania

**Keywords:** electrohydrodynamic printing, antibiotic patches, drug release, polymer, bacterial cellulose

## Abstract

This study investigates the usage of electrohydrodynamic (EHD)-3D printing for the fabrication of bacterial cellulose (BC)/polycaprolactone (PCL) patches loaded with different antibiotics (amoxicillin (AMX), ampicillin (AMP), and kanamycin (KAN)) for transdermal delivery. The composite patches demonstrated facilitated drug loading and encapsulation efficiency of drugs along with extended drug release profiles. Release curves were also subjected to model fitting, and it was found that drug release was optimally adapted to the Higuchi square root model for each drug. They performed a time-dependent and diffusion-controlled release from the patches and followed Fick’s diffusion law by the Korsmeyer–Peppas energy law equation. Moreover, produced patches demonstrated excellent antimicrobial activity against Gram-positive (*Staphylococcus aureus*) and Gram-negative (*Escherichia coli*) strains, so they could be helpful in the treatment of chronic infectious lesions during wound closures. As different tests have confirmed, various types of antibiotics could be loaded and successfully released regardless of their types from produced BC/PCL patches. This study could breathe life into the production of antibiotic patches for local transdermal applications in wound dressing studies and improve the quality of life of patients.

## 1. Introduction

Proper wound management is essential to guard wounded areas from pathogens responsible for secondary infections. For example, if germs rapidly multiply in a wound, and the infection spreads, it could cause a serious internal infection [1]. Moreover, an internal infection can delay wound healing by extending the inflammatory stage while the immune system responds and clears the infection [2]. In this respect, antibiotic patches are an innovative approach, i.e., to deliver improved healthcare, reduce overall healthcare costs, increase drug effectiveness, and balance the toxicity of drugs involved in local wound management [3]. They are essential in managing local infections where high concentrations of antibiotics are required locally [4]. On the other hand, their concentrations in the patches should be kept under control as high amounts of antibiotics can lead to systemic toxicity [5]. Beta-lactam antibiotics (amoxicillin, ampicillin) and aminoglycosides (kanamycin) are well-known drugs applied to inhibit bacterial spread in wound dressing applications [6]. Their loadings into transdermal patches for local wound dressing applications can provide beneficial antibacterial activity while being absorbed into the body. Furthermore, local administration can limit the potential for systemic absorption of antibiotics, diminishing antibiotic resistance [6].

Drug delivery systems have been extensively explored in recent decades to provide more effective and side effect-free treatments for patients suffering from diseases. The most common forms of drug delivery systems include oral tablets, liquid injections, microspheres, hydrogels, liposomes, micelles, and nanoparticles [7]. However, such delivery systems have challenges in maintaining precise control of the rate and duration of drug release, require repeated administration, and have long-term release problems [8,9,10,11,12,13]. To overcome these difficulties, drug embedded thin polymer patches have been proposed as a versatile approach to prolong drug release, and they are currently being widely used [14]. Thin polymer patches are flexible and can be produced in various shapes and sizes [15]. Three-dimensional (3D) printers, such as inkjet printers and thermal inject printers, can create small-sized patches (with high placement accuracy) using thermal or mechanical actuating mechanisms [16]. However, inkjet printing is limited in writing operations that involve small micrometers, and cannot be utilized for drug reservoirs requiring several micrometers [17]. The electrohydrodynamic (EHD)-3-dimensional (3D) printing method is capable of achieving micrometer-scale, complex, high-resolution 3D structures, in ambient temperature operations, with single-step productions [18]. The EHD uses an electric field on the polymer solution using micro/nano-scaled nozzle tips [19]. The EHD-3D printing process is the same as the conventional EHD process, yet the sample is collected close to the needle, which is quite small in diameter. Therefore, it is possible to achieve higher print resolution without replacing the nozzle tip with a smaller diameter nozzle, using highly viscous colloidal solutions, or viscous polymeric fluids [20]. Moreover, the EHD-3D printing method can provide essential leads for cell alignment and cell contact guidance, for better cell proliferation and tissue formation, with optimal porosity, inter-fibrous pore size, and pore distribution [21,22,23]. The dosage of the antibiotics can be adjusted according to the patient’s individual needs by altering the size of the EHD-3D printed scaffolds and the content of the solid materials. In addition, through EHD-3D printing, the formulation can be achieved during the production and purification of the drugs, improving efficiency, and decreasing the costs for pharmaceuticals industries.

Polymeric biomaterials and composites are widely used in the EHD-3D printing method due to their polymerizing capabilities, by various methods, to make them “3D printable”. They provide physical support for cell attachment, but they also offer transport of therapeutic agents, such as drugs, proteins, growth factors, and chemical agents [24]. PCL is one of the biomaterials that has semi-crystalline, biocompatible, and biodegradable features, with ideal mechanical properties and biological performance [25]. The usage of PCL in drug dosage forms is quite common due to its ability to be easily absorbed into the porous structure of the drug active substance, its capability of returning the active substance to the body at high rates, its complete resorption from the body, and its extended bioavailability on the tissue [26]. The high biocompatibility and slow(er) biodegradation rate, as well as the required flexibility of PCL, make it applicable for use in local transdermal applications. Furthermore, PCL patches show great potential for the delivery of therapeutics, and they can be combined with other biomaterials, such as bacterial cellulose (BC). BC is a biomaterial that shares common features with extracellular matrix (ECM) components [27]. Its porosity can provide the release of antimicrobial agents, drugs, and other bio-functional materials to the targeted tissue in a controlled manner [28,29]. Blending this remarkable polymer with PCL can offer improved cell viability rates while an antibiotic patch releases its therapeutic agent [30]. Moreover, thanks to the hydrophilic nature of BC, moisture can be maintained, and dehydration of the wound and crust formation can be prevented.

This research aims to produce layer-on-layer stacked antibiotic patches from BC/PCL blend polymers carrying various broad-spectrum antibiotics using the EHD-3D printing method, and compare their releasing properties with drug release studies and drug release kinetic models for local transdermal applications in wound dressing applications. The use of these antibiotics will exhibit the versatility of the antibiotic patches in terms of different antibiotic loadability.

## 2. Materials and Methods

### 2.1. Materials

Polycaprolactone (PCL, Mw 80,000 g/mol), dimethylformamide (DMF), and dichloromethane (DCM) were purchased from Sigma-Aldrich (St. Louis, MO, USA). Bacterial cellulose (BC) pellicle was provided by the Department of Medical Microbiology, Medipol University (Istanbul, Turkey). Amoxicillin (AMX, Mw 365.4 g/mol), ampicillin (AMP, Mw 349.4 g/mol), kanamycin (KAN, Mw 600.6 g/mol), and phosphate buffered saline (PBS, pH 7.4) were also obtained from Sigma-Aldrich (Steinheim, Germany).

### 2.2. Preparation of Solutions

BC pellicle was sonicated in DMF for 1 h using a sonicator (Branson Sonifier 250, BRANSON, Danbury, CT, USA) at a power output of 100% and then was centrifuged at 6000 rpm for 600 s. After each centrifugation step, the supernatant was removed and, finally, fragmented BC was obtained with almost no DMF residue. After that, 10 wt.% PCL solution was prepared in DCM with continuous magnetic stirring (WiseStir^®^, MSH-20A, Germany) for 24 h at 23 °C. As the last step, fragmented BC was blended with 10 wt.% PCL solution (5:95 wt. ratio), and the final blend was magnetically stirred for 1 h at an ambient temperature (23 °C).

The direct blending of the drug with a polymeric solution is the simplest method for fabricating drug-containing patches through the processing of a polymeric [31]. Therefore, three different types of antibiotics (AMX, AMP, and KAN) were individually blended with BC/PCL solution under constant stirring at an ambient temperature (23 °C) for 12 h prior to EHD-3D printing. The initial loading of each drug was 50 mg/mL. After blending, final solutions were stirred at an ambient temperature (23 °C) for another 12 h before EHD-3D printing. Prepared solutions were measured for their viscosity, density, electric conductivity, and surface tension using a Brookfield DV-E programmable viscometer, a standard density bottle (5 mL), a KRÜSS K100SF tensiometer, and a WTW Cond-3110-SET1 conductivity meter, respectively. The measurements were carried out in triplicate at the ambient temperature of 23 °C and relative humidity of 50 ± 3%.

### 2.3. EHD-3D Printing

**Figure 1:**
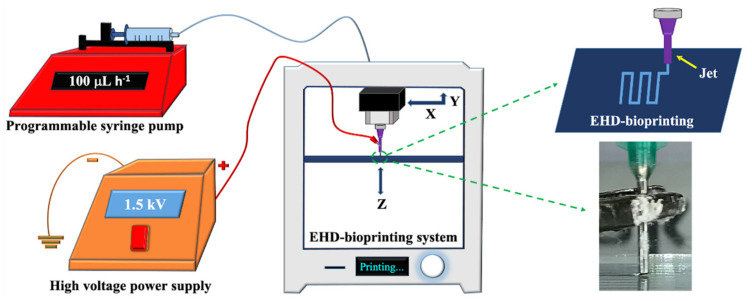
Experimental EHD-3D printing setup with a closer view of the EHD-3D patterning.

The EHD-3D printing was accomplished by using an altered lab system, which consisted of an X–Y stage motion control head, a glass substrate holder, a nozzle holder with Z-axis control, a high voltage power supply (output DC voltage from 0 to 40 kV), and a solution feeding system (Figure 1). The solution feeding system consisted of a programmable syringe pump (NE-1000, New Era Pump System Inc., Farmingdale, NY, USA) and a stainless steel nozzle (with 21G orifice). The syringe was filled with a prepared solution, which gave a constant solution source through the EHD-3D printing process.

For EHD-3D printing, SolidWorks software was used to design the square patches as 10 x 10 mm^2^, and layers were piled in 5 min with a 90°angle as pre-designed. The distance between the nozzle and substrate was stabilized to 1 mm for experimental setup after the preliminary investigations [23,30]. According to the study, as mentioned earlier, the flow rate was optimized for reproducible and uniform fiber formation to 100 mLh^−1^ for all samples. EHD-3D printed microfibers were deposited using 1.5 kV and stacked with 0°/90° angles as two layers. Upon completing the EHD-3D printing process, drug patches were removed from the substrate. The vacuum oven was used to evaporate residue solvents that may have remained in the samples before characterization tests. Finally, yield percentages of samples were calculated using Equation (1) and all calculations were repeated three times:

Yield% = 100 × (weight of collected product/weight of solid materials applied)(1)

### 2.4. Characterization of Drug Patches

Morphological characterization of the neat and the drug-containing patches was investigated by a ZEISS MA/EVO10 scanning electron microscope (SEM). Samples were placed on microscopic glass slides and mounted on a specimen stub with double-sided carbon tape. Then, they were sputter-coated with Au for 60 s using a Quorum SC7620 Mini Sputter Coater before SEM imaging. After SEM imaging at an acceleration voltage of 10 kV, diameters of the individual fibers, inter-fibrous pore sizes, and porous sizes and porosity on the fibers within the EHD-3D printed patches were measured using ImageJ.

A JASCO FT/IR-4000 Fourier transform infrared spectrometer (FTIR) was used to investigate the chemical characterization of the EHD-3D printed patches. Each FTIR spectra was taken at 4 cm^−1^ resolution over the range of 4000–400 cm^−1^.

Uniaxial tensile tests were performed using an INSTRON 4411 tensile device (Instron, Norwood, MA, USA) with a 50 N load cell under a crosshead speed of 5 mm/min at the ambient temperature (23 °C). All patches were subjected to tensile testing. Each measurement was taken in triplicate, and the ultimate tensile strength and Young’s modulus values were calculated from the stress–strain curves of the tested samples.

The swelling characteristics of the EHD-3D printed patches were measured according to Equation (2) after the samples were submerged in PBS at 37 °C for 24 h:

Degree of swelling (%) = 100 × (M − Md)/Md(2)

where M is the weight of each sample after submersion in the PBS solution for 24 h and Md is the weight of the sample after submersion in the buffer solution for 24 h in its dry state.

Prior to AMX, AMP, and KAN drug loading (DL) and encapsulation efficiency (EE) studies, all drug-containing patches were utterly dissolved in DCM. Thereafter, AMX, AMP, and KAN concentrations in DCM were determined using UV Spectrophotometer (UV1280, Shimadzu, Kyoto, Japan) at 229, 230 and 207 nm. The DL and EE percentages of AMX, AMP, and KAN in patches were calculated using Equations (3) and (4) [31]:

DL (%) = 100 × (Weight of antibiotic entrapped in the sample/weight of the patch)(3)

EE (%) = 100 × (Determined drug content/theoretical drug content)(4)

### 2.5. Drug Release Studies

The in vitro release kinetics of antibiotics from EHD-3D printed patches in PBS medium were determined using a UV Spectroscopy by recording the wavelengths at 229, 230 and 207 nm for AMX, AMP, and KAN, respectively. A linear calibration curve was established based on standard solutions with concentrations ranging from 5–200 μg/mL. The amount of the drug present in a patch was calculated from the obtained calibration curve for each drug. All drug-release experiments were carried out using the total immersion method with a known quantity of sample immersed in a sealed vial bottle containing 20 mL of PBS and incubated in a shaker at a constant incubation temperature 37 °C. At designated time intervals ranging between 0 and 14 days (336 h), a 1 mL release medium was taken out and replenished with an equal volume of fresh PBS. Supernatants were filtered using 0.45 μm Millipore. The amount of the drugs in the withdrawn release mediums was determined at the same wavelengths previously mentioned against the pre-determined calibration curves for each AMX, AMP, and KAN drugs using the UV spectrophotometer. The obtained data were used for assessing cumulative release and mathematical modeling of release mechanisms.

### 2.6. Application of Drug Release Data to Mathematical Models

The application of drug release data to mathematical models is usually a few mathematical equations that define the dissolution profile. Once a suitable function has been selected, an assessment of the dissolution profile can be performed, and the drug release profile can be correlated with drug release kinetic models.

#### 2.6.1. Zero Order Kinetics

Zero order kinetics describe the process of continuous drug release from a drug delivery system, and the level of the drug in the blood remains constant throughout the drug release. Hence, the drug release kinetics from an in vitro release study are plotted against time to study the data. In this kinetic model, the drug release rate is independent of its concentration. Used zero order equation (Equation (5)) is [32]:

C_t_ = C_0_ + K_0_t (5)

In the equation, C_t_ shows the amount of drug released in a given time, C_0_ is the initial concentration of the drug, and K_0_ is the zero order rate constant.

#### 2.6.2. First Order Kinetics

First order kinetics describe a concentration-dependent drug release rate from the delivery system. The used first order equation (Equation (6)) is [32]:

dC/dt = K_1_(C_t_ − C_0_) (6)

In the Equation (6), dC/dt shows the rate of change in concentration with respect to time, and K_1_ represents the first order rate constant expressed in units of time (t).

#### 2.6.3. Higuchi Model

The Higuchi model describes the release of drugs from the insoluble matrix as a square root of a time-dependent process based on Fickian diffusion. The used Higuchi equation (Equation (7)) is [32]:

Q = K_H_t^1/2^ (7)

In the Equation (7), Q represents the cumulative amount of drug released at the time (t per unit area), K_H_ shows the Higuchi dissolution constant.

#### 2.6.4. Korsmeyer–Peppas Model

In order to understand the mechanism of drug diffusion from patches, release data were detected using the well-known empirical equation proposed by Korsmeyer and Peppas. The Korsmeyer–Peppas model describes the drug release mechanism from a polymeric system. The used Korsmeyer–Peppas equation (Equation (8)) is [33]:

M_t_/M∞ = K_kp_t*^n^* (8)

In the Equation (8), M_t_ represents the amount of drug released at time t, M∞ shows the amount of drug released after ∞ period, *n* symbolize the release exponent, and K_kp_ depicts the Korsmeyer–Peppas release rate constant.

For a cylindrical geometry with a non-swellable drug release system, when n = 0.45, the drug release mechanism is Fickian diffusion, when *n* = 1, Case II transport occurs leading to the zero-order release and when the value of n is between 0.45 and 1, Non-Fickian diffusion occurs [33].

### 2.7. Antibacterial Activity Assay

The inhibition method zone was used to investigate the antibacterial activity of the drug patches against Gram-positive (*S. aureus*) and Gram-negative (*E. coli*) bacterial strains as the model organisms. The bacterial cultures from different individual colonies were cultivated in Luria-Bertani (LB) broth (10 g/L bacto-tryptone, 5 g/L yeast extract, 5 g/L NaCl) overnight at 37 °C. The bacterial strains (107 colony-forming unit (cfu)/mL) were inoculated on LB-agar (LB broth with 15 g/L agar) plate using sterile cotton swabs. The equal weight of the patches was gently placed on the bacteria inoculated agar plates and incubated at 37 °C for 24 h. Zones of inhibition of the patches were evaluated by measuring the clear area that formed around each patch. Experiments were repeated in triplicate, and the average diameter of zones of inhibition was measured.

## 3. Results and Discussion

### 3.1. Characterization of Fabricated Samples

The EHD systems are governed by processing parameters and the properties of the working polymer solution [34]. EHD-3D printing is a single-step production process that provides both nano and micro-scale writing and combines the electrohydrodynamic atomization and 3D writing methods. All of the properties of the working polymer solution, including viscosity, electrical conductivity, density, and surface tension, are given in Table 1. The presence of the drugs in the resulting patches did not cause any significant influence on the physical properties of the BC/PCL solutions.

**Figure 2:**
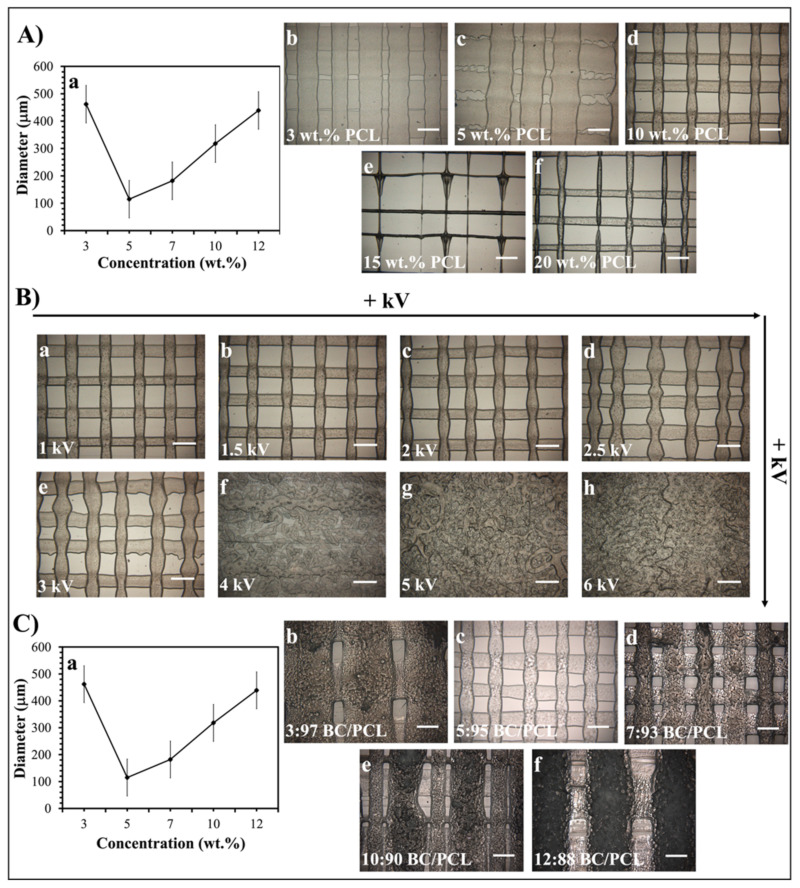
(**A**) EHD-3D printed fiber diameter graph (a) using different concentrations of PCL solution and optical images of the patches (b–f). (**B**) Optical images of the various voltage value (1–6 kV) applied EHD-3D printed deposited fibers of 10 wt.% PCL with different alignments and structures. (**C**) EHD-3D printed fiber diameter graph (a) using different concentrations of BC/PCL composition and optic images of the samples (b–f). (All scale bars indicate 100 µm).

As stated previously [23], the EHD-3D printing process could be influenced by the concentration of the printed solution and the electric field strength. In this study, the diameters of resultant fibers changed with variant PCL solution concentration values under 1.5 kV, and 10 wt.% PCL found to be the best sample with 100 ± 5 µm mean fiber diameter (Figure 2A). Additionally, different voltage values from 1 to 6 kV were applied for detecting the optimal fiber structure of 10 wt.% PCL (Figure 2B). Consequently, 1.5 kV was found to be the optimal value for EHD-3D printing of 10 wt.% PCL solution. In another preliminary study [30], different ratios of BC (3, 5, 7, 10, and 12 wt.%) were mixed with 10 wt.% PCL to produce layer-on-layer stacked patches. According to optical microscopy images, the 5:95 (wt. ratio) BC/PCL solution sample showed the best fiber structure and inter-fibrous pore size with 100 × 100 μm^2^ (Figure 2C). Therefore, BC/PCL 5:95 (wt. ratio) blend solution was selected to carry AMX, AMP, and KAN antibiotics for further research made in this study. 

**Figure 3:**
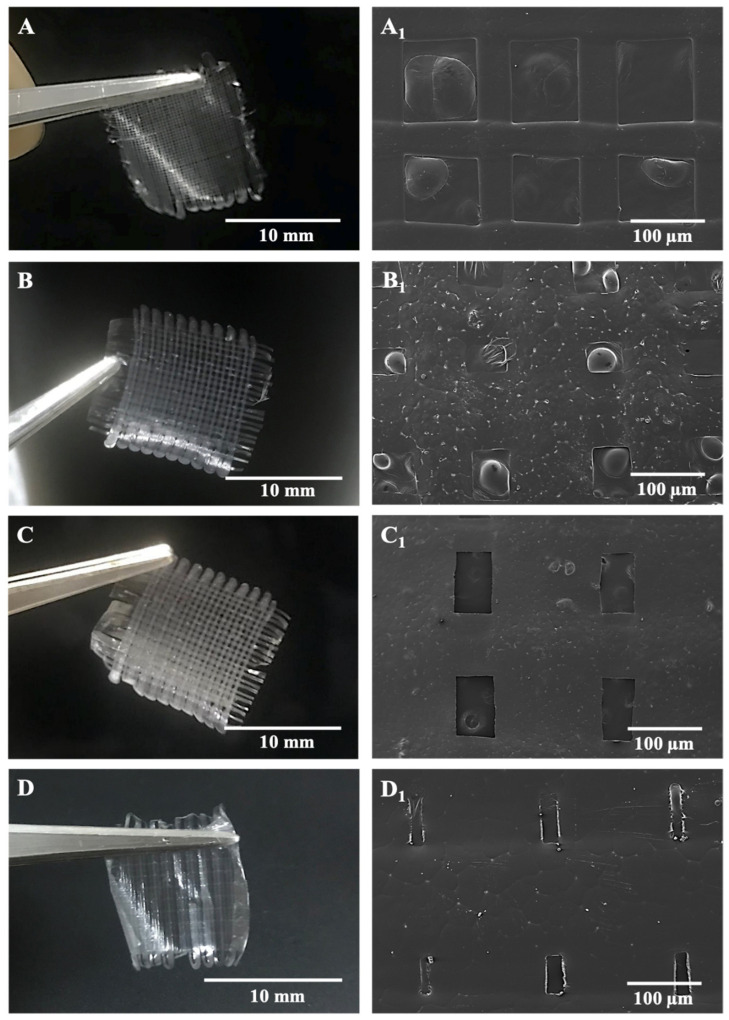
SEM images of EHD-3D printed patches with digital photographs of BC/PCL 5:95 (wt. ratio) patch with inter-fibrous pore size 100 × 100 μm^2^ (**A**,**A_1_**), AMX-loaded patch with inter-fibrous pore size 100 × 100 μm^2^ (**B**,**B_1_**), AMP-loaded patch with inter-fibrous pore size sub-100 × 100 μm^2^ (**C**,**C_1_**) and KAN-loaded patch with inter-fibrous pore size sub-100 × 100 μm^2^ (**D**,**D_1_**).

Microfibers in the antibiotic patches were pictured with SEM after being left in the vacuum oven and represented along with digital photographs of relevant samples in Figure 3. According to morphological observations on SEM and measurements in the ImageJ program, inter-fibrous pore sizes were 100 × 100 μm^2^ for BC/PCL (5:95 wt. ratio) and BC/PCL+AMX samples and sub-100 × 100 μm^2^ for BC/PCL+AMP and BC/PCL+KAN samples, with mean fiber diameters of 50.1 ± 0.7 μm for BC/PCL, 77.1 ± 4.8 μm for BC/PCL+AMX, 86.3 ± 45.8 μm for BC/PCL+AMP, and 140.2 ± 9.1 μm for BC/PCL+KAN, respectively.

The variation in fiber diameter can be explained due to the varied molecular weight difference caused by different drugs since high molecular weight promotes obtaining fibers with a larger diameter [35]. Thus, the EHD-3D printing yields thinner fibers when the molecular weight decreases. However, the AMP drug had more molecular weight than AMX and BC/PCL+AMX fibers were thinner in diameter than BC/PCL+AMP fibers. In this case, this phenomenon can be explained based on the chemical structure of the monomer unit of BC/PCL 5:95 (wt. ratio) and its chemical interaction with AMP antibiotic. Therefore, FTIR tests were performed to examine the presence and possible chemical interaction between polymers and antibiotics.

SEM images of patches revealed the interconnected pores on the microfibers. The rapid evaporation of DCM may have created a rise in local phase separation, and the solvent-rich regions may have transformed into pores during the process [36]. In this study, pore sizes were in the micro-range, and porosity varied with different types of antibiotics, as shown in Table 2. As a general rule, the pore size and porosity increase as a consequence of the addition of the antibiotics; the highest increase being recorded for the patches with AMX (highest pore size—10.20 ± 0.35 µm^2^) and AMP (highest porosity—53.21 ± 1.76%) during the patch with KAN exhibit the lowest increase.

Biomedical applications require highly porous patches that should mimic the biological environment they are intended to interact with to improve biocompatibility and allow increased drug loading capacity [37]. Biological environments usually have porous structures ranging from nano- to macroscale [38].

**Figure 4:**
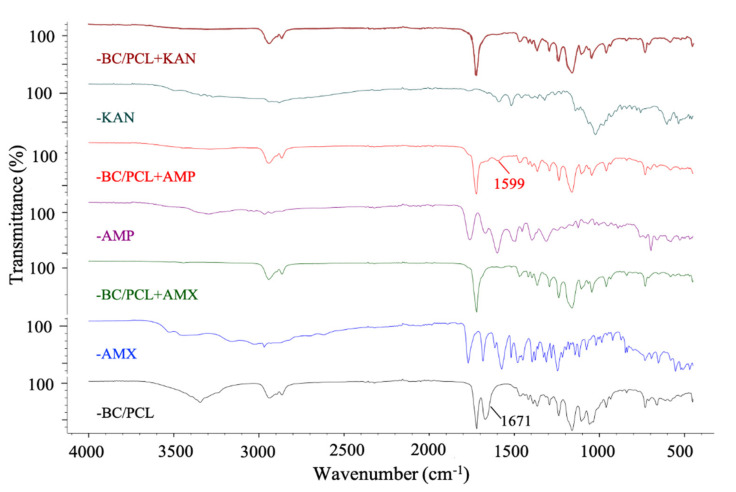
FTIR spectra of EHD-3D printed patches.

FTIR spectroscopy was used to confirm the possible interaction between antibiotics and polymers in produced patches. Figure 4 shows the FTIR absorbance spectra of BC/PCL 5:95 (wt. ratio), neat AMX, BC/PCL+AMX, neat AMP, BC/PCL+AMP, neat KAN, and BC/PCL+KAN samples. Typical BC and PCL absorption bands were observed in all samples [30,39]. When the functional groups of the drug and the polymer interact chemically, FTIR spectra is expected to show band shifts and dilatations in the spectrum compared to the pure drug and polymer spectra [40]. No chemical binding between AMX and BC or PCL was observed when examining the AMX containing BC/PCL (5:95 weight ratio) patch. In addition, no chemical interaction between KAN and BC/PCL (5:95 weight ratio) was observed in the BC/PCL+KAN patch. AMX or KAN drugs were not chemically bound to the hydrogen bonds of BC or the carbonyl groups of PCL. They were physically bounded to the patches of the BC/PCL (5:95 weight ratio) blend. A physically bounded drug can be efficiently released from the patch. It is expected to have a faster release. Thus, a burst, such as a release, and high initial antimicrobial activity is expected [41].

On the other hand, the band of BC/PCL at 1671 cm^−1^ shifted to 1599 cm^−1^ in BC/PCL+AMP patch, and this chemical interaction can be the reason for the increased fiber diameter of the BC/PCL+AMP patch. Moreover, solution characteristics can also be the reason for this, even though the AMP has a higher molecular weight than AMX. This difference can be mainly associated with the chemical structure, AMP being a very soluble molecule, oligosaccharide (Mw = 484.499 g/mol) with six free hydroxyl and four free amino groups, which can strongly interact via hydrogen bonds with the polymeric patch.

**Figure 5:**
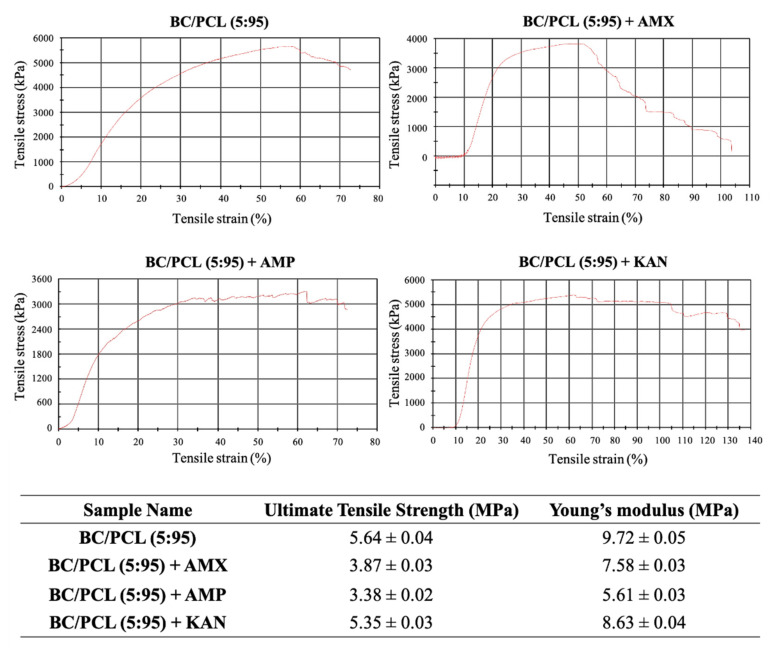
Stress-strain curves of EHD-3D printed patches with ultimate tensile strength and Young’s modulus values.

Mechanical properties of EHD-3D printed patches (BC/PCL, BC/PCL+AMX, BC/PCL+AMP, and BC/PCL+KAN) were investigated using a tensile testing device, and relevant stress–strain curves for patches are represented in Figure 5. Ultimate tensile strength and Young’s modulus of the BC/PCL patch were 5.64 and 9.72 MPa, respectively. AMX, AMP, and KAN loaded BC/PCL patches exhibited lower ultimate tensile strength (3.87, 3.38, and 5.35 MPa, respectively) than the BC/PCL patch. As stated previously [42], poor interface interaction and unstable phase dispersion can compromise the mechanical properties of polymer composite systems, which can be the reason behind the reduction in tensile strength with the addition of antibiotics to the BC/PCL blend. AMX, AMP, and KAN contain patches that also exhibited poor Young’s modulus values (7.58, 5.61, and 8.63 MPa, respectively) compared to the BC/PCL patch. As it is known, a decrease in Young’s modulus results from an increase in the porosity of that material [43]. In this study, porosity values of AMX, AMP, and KAN loaded patches are found higher than the BC/PCL patch and this increase in porosity reduced Young’s modulus values. The Young’s modulus of the human soft tissues ranges from 0.6 to 1.7 MPa [44]. Hence, it is thought that the produced drug patches can meet the mechanical requirements needed at various anatomical locations, such as the skin, muscle, or tissues where the drug needs to be delivered.

**Figure 6:**
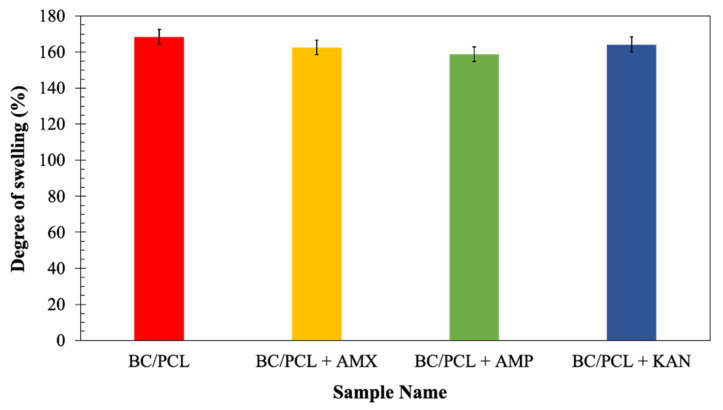
The degree of swelling (%) of EHD-3D printed patches.

Since swelling properties of a scaffold are vital because of its role in enabling liquid absorption of physiological secretions and allow the efficient exchange of nutrients and waste of regenerated tissue [34], the neat and the drug-containing patches were further characterized to determine their swelling behavior after submersion in the phosphate buffer saline (PBS, pH = 7.4) at 37 °C for 24 h. At the end of 24 h of immersions, the degree of swelling of the BC/PCL patch (168%) was comparable with those of the patches loaded with antibiotics: AMX loaded patch ≈ 162%, AMP loaded patch ≈ 159%, and KAN loaded patch ≈ 164% (Figure 6). Considering a release rate of about 30–35% of the antibiotics, it can assume that the presence of antibiotics increases the swelling ratio because of the additional porosity assured after the antibiotic release. The swelling and water uptake depends on the hydrophobicity of the polymer [45], as it is well-known that the PCL is a hydrophobic polyester and can prevent water uptake [46]. Besides, since BC is a hydrophilic polymer, it can pick the water that diffuses into the PCL matrix while increasing the equilibrium swelling of the water [47].

Antibiotic addition to the BC/PCL blend in the selected quantities did not interfere with patch water uptake capability. Consequently, produced patches can prevent loss of body fluid and nutrients due to the enhanced swelling ability while releasing their loaded drugs.

The yield percentage, drug loading, and antibiotic encapsulation efficiency of the patches are presented in Table 3. The drug loading was similar for the three different antibiotics (21%), while the encapsulation efficiency of the drugs was similar for the three different antibiotics and between 96% and 98%.

### 3.2. Drug Release and Mathematical Modeling of Release Mechanisms

**Figure 7:**
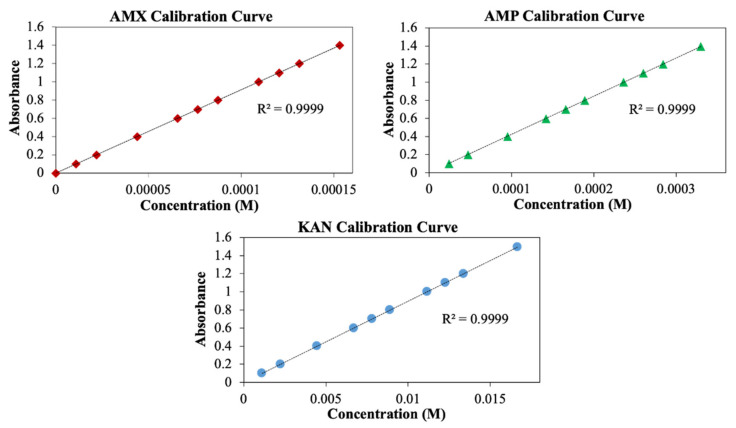
Calibration curves for AMX, AMP, and KAN drugs.

Measurement of drug release from the patches was performed using the UV spectroscopy method. For this purpose, AMX, AMP, and KAN calibration curves were prepared to determine release kinetics (Figure 7).

**Figure 8:**
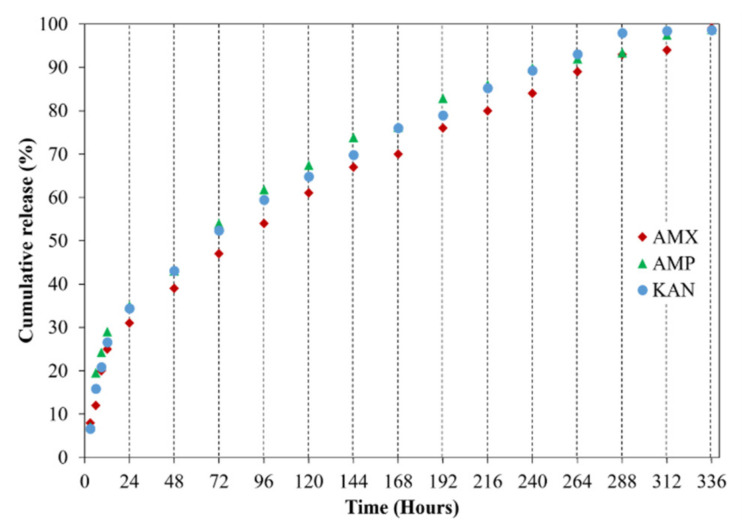
Cumulative release (%) of loaded antibiotic patches.

It is crucial to investigate drug release profiles for several days due to the advantages of increasing medication and drug efficacy, reducing side effects, and reducing the frequency of administration of these drugs [48]. In this study, the release of AMX, AMP, and KAN drugs from EHD-3D printed patches were examined for 14 days (336 h) in PBS, pH 7.4 (Figure 8).

All release curves mainly consisted of two phases as the first phase, a “burst release”, and then “slow-release”, so that the second phase took place. The initial burst release is beneficial for releasing antibiotic drugs since it is essential to remove the spreading bacteria before they start to multiply [49]. Hence, to prevent the remaining population for several organisms that survive the first immediate release, followed by a sustained release of antibiotics is required [50]. After the burst release phase, increases were observed in the release of drugs due to the concentration of AMX, AMP, and KAN in the patches, and then these increases were found to be linear and prolonged over many days. The AMX loaded patch showed 99.1 ± 0.6% cumulative release after 336 h, whereas the cumulative release of AMP was about 98.1 ± 0.3% after 336 h. The cumulative release of KAN was 98.6 ± 0.6% after 336 h of release studies. The increased surface area for the release of the drugs can be responsible for improving the release of the drugs when formulated as microfibers [51].

Wang et al. [52] produced antibiotically loaded (tetracycline hydrochloride) patches using polycaprolactone (PCL), polyvinyl pyrrolidone (PVP), and their composite system (PVP-PCL) by the EHD-3D printing method. The researchers examined the release of antibiotic drug from PCL-PVP dosage forms for 5 days. They witnessed a slower antibiotic release compared to neat PCL or PVP. It was observed that the size of gaps between the fibers in the printed patches influenced the release of the antibiotic material. As a result of this study, it was found that the EHD-3D printing method is a promising method for adapting the dosage forms with minimum adjuvants and large-scale procedures.

**Figure 9:**
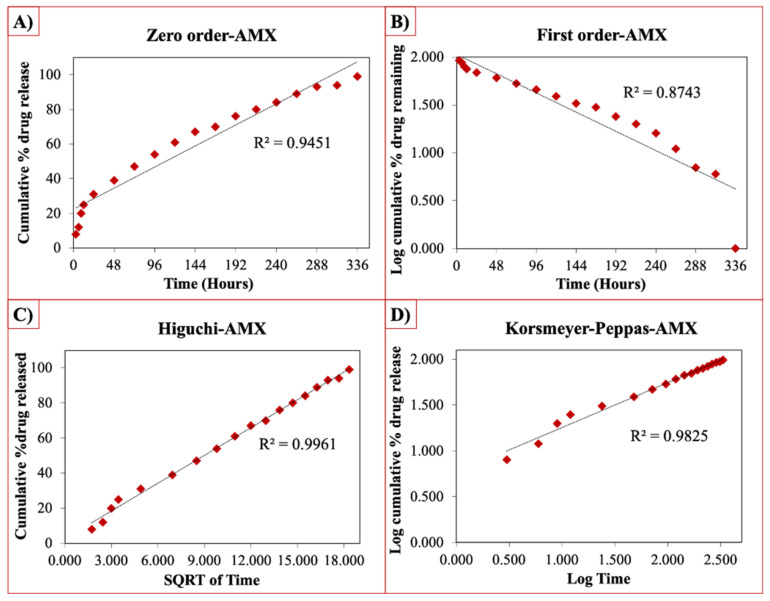
Zero order (**A**), first order (**B**), Higuchi (**C**), Korsmeyer–Peppas (**D**) kinetic releases of BC/PCL+AMX patch.

**Figure 10:**
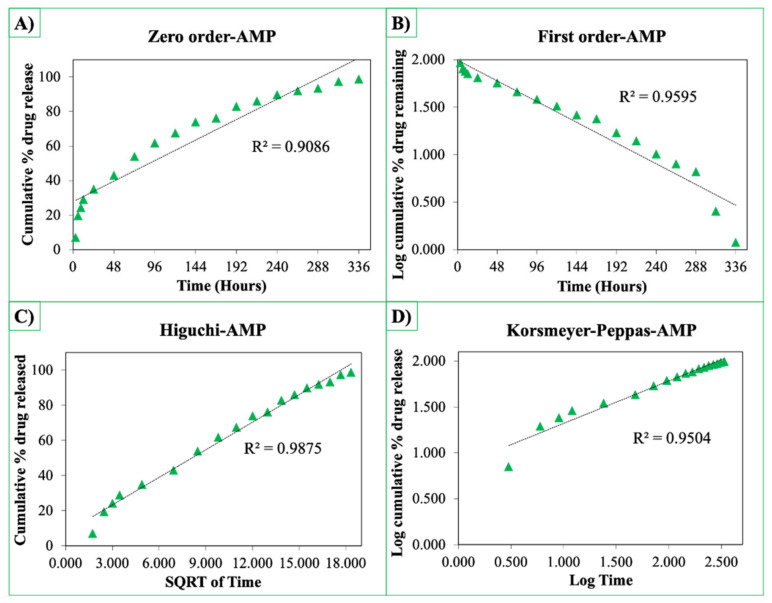
Zero order (**A**), first order (**B**), Higuchi (**C**), Korsmeyer–Peppas (**D**) kinetic releases of BC/PCL+AMP patch.

**Figure 11:**
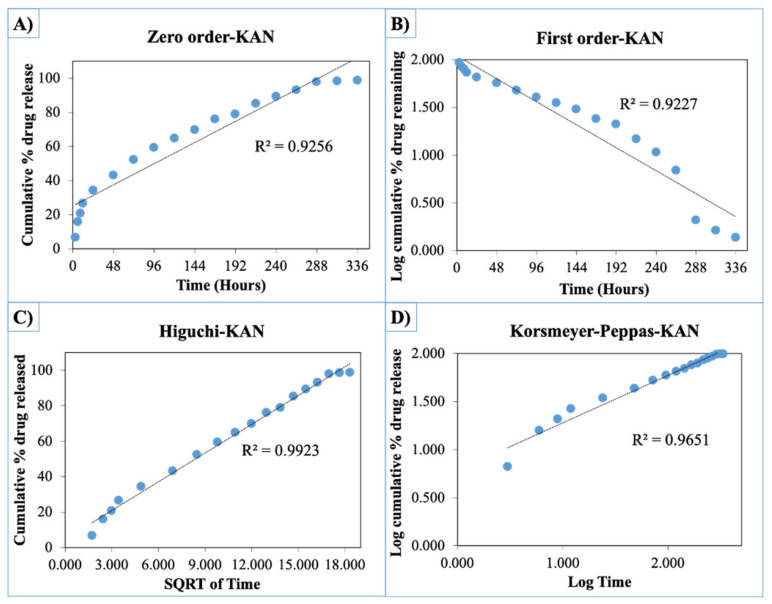
Zero order (**A**), first order (**B**), Higuchi (**C**), Korsmeyer–Peppas (**D**) kinetic releases of BC/PCL+KAN patch.

The release curves were also subjected to model fitting consisting of various models such as zero order, first order, Higuchi, and Korsmeyer–Peppas models (Figure 9, Figure 10 and Figure 11). Graphical representations of the cumulative percentage of drug release against time showed that the release of AMX, AMP, and KAN from the patches correctly followed the Higuchi model. Drug release profiles were very close to the trend line or regression line and had the highest coefficient value (R^2^) for AMX, AMP, and KAN loaded patches (0.9961, 0.9761, and 0.9888, respectively).

The Korsmeyer–Peppas model was applied to release profiles to determine the diffusion types. After the Korsmeyer–Peppas model application, the diffusional exponent, which is indicative of the release mechanism (n), was found for all samples. The *n* = 0.45 value indicates Fickian diffusion, while values between 0.45 and 1.00 suggest non-Fickian transport and values higher than 1.00 suggest zero order/case II transport of a drug [53].

The diffusion exponent ‘n’ was 0.49, 0.46, and 0.48 for AMX, AMP, and KAN loaded patches, respectively. The ‘n’ values for AMX, AMP, and KAN loaded patches indicated that the releases of the drugs followed Fick’s law of diffusion. As a general conclusion, regardless of the solubility of the antibiotic, the release of the drugs from patches was likely to be controlled by a diffusion mechanism [54].

### 3.3. Antibacterial Assessment of Fabricated Patches

The antibacterial potentials of the patches were evaluated as the diameter of the zone around the 3D printed scaffolds. The experiment was performed in triplicate, and the average of the zones of inhibition used to determine the antibacterial activity.

Table 4 shows the inhibition zones after 24 h of incubation. The inhibition zones were observed as a clear circular area surrounding the samples specifying the zone of bacteria that was destroyed or prevented from growing. BC/PCL was used as a negative control and did not have any antibacterial properties, as they showed no activity against the two bacterial strains (*S. aureus* and *E. coli*). According to the results in Table 4, BC/PCL+AMX, BC/PCL+AMP, and BC/PCL+KAN drug patches showed 43, 39, and 30 mm diameter inhibition zones against *S. aureus*, respectively. On the other hand, the same drug patches showed 35, 37, and 35 mm inhibition zones against *E. coli*, respectively. Results suggest that all three different antibiotics (AMX, AMP, and KAN) containing patches can be used as an effective antibacterial patch on both Gram-positive and Gram-negative bacteria.

## 4. Conclusions

Antibiotic patches composed of BC/PCL, AMX, AMP, and KAN, were prepared by the EHD-3D printing method, resulting in aligned layer-on-layer stacked fibers. The drug release properties and drug release kinetic models of different antibiotics loaded patches were evaluated. Drug-containing patches displayed micron-scaled fibers and inter-fibrous pore size. Moreover, microfibers exhibited a porous structure, which provided improved biocompatibility and increased drug loading capacity. All patches displayed enhanced swelling ability. They were found able to meet mechanical requirements needed at various anatomical locations according to tensile test results. The release of AMX, AMP, and KAN antibiotics from BC/PCL dosage forms was shown at 14 days (336 h). The kinetic models (zero order, first order, Higuchi, and Korsmeyer–Peppas) on drug release were profiled for each antibiotic patches. It was found that the release of the drugs from the patches followed Fick’s law of diffusion. The antibacterial potential was evaluated, and AMX, AMP, and KAN were found effective on both Gram-positive and Gram-negative bacteria to provide antimicrobial activity to BC/PCL patches. It is suggested that the single-step EHD-3D printing method offers rapidly tailored dosage forms with minimal excipients. Moreover, various types of antibiotics could be loaded and successfully released, regardless of their types, from produced BC/PCL patches. Produced antibiotic patches can meet the characteristic requirements needed in local transdermal applications with accelerated release characteristics, adjustability, and versatility for wound dressing studies, and decrease the pharmaceuticals industries’ costs.

## Figures and Tables

**Table 1:** Physical properties of EHD-3D printed solutions. (Mean ± SD, *n* = 3).

Sample Name (wt. ratio)	Viscosity (Pa.S)	Electrical Conductivity (µ.S cm^−1^)	Density (kg m^−3^)	Surface Tension (mN m^−1^)
BC/PCL (5:95)	363 ± 0.5	1.20 ± 0.03	1445 ± 2	52.8 ± 0.1
BC/PCL (5:95) +AMX	368 ± 0.3	1.20 ± 0.02	1448 ± 1	52.9 ± 0.1
BC/PCL (5:95) +AMP	371 ± 0.1	1.20 ± 0.02	1452 ± 1	53.2 ± 0.5
BC/PCL (5:95) +KAN	376 ± 0.2	1.20 ± 0.01	1459 ± 1	53.4 ± 0.3

**Table 2:** Pore size and porosity of EHD-3D printed patches. (Mean ± SD, *n* = 3).

Sample Name (wt. ratio)	Pore Size (µm^2^)	Porosity (%)
BC/PCL (5:95)	2.22 ± 0.85	11.52 ± 1.14
BC/PCL (5:95) +AMX	10.20 ± 0.35	32.43 ± 1.36
BC/PCL (5:95) +AMP	6.18 ± 0.12	53.21 ± 1.76
BC/PCL (5:95) +KAN	3.06 ± 1.09	19.11 ± 1.44

**Table 3:** Yield percentage, drug loading, and encapsulation efficiency of the patches. (Mean ± SD, *n* = 3).

Sample Name (wt. ratio)	Yield (%)	Drug Loading (%)	Encapsulation Efficiency (%)
BC/PCL (5:95)	98.4 ± 0.6	-	-
BC/PCL (5:95) +AMX	98.2 ± 1.3	21.9 ± 0.3	97.4 ± 0.9
BC/PCL (5:95) +AMP	96.3 ± 2.1	21.7 ± 0.7	96.8 ± 1.6
BC/PCL (5:95) +KAN	97.9 ± 1.9	21.9 ± 0.5	98.6 ± 1.4

**Table 4:** Antibacterial properties of the EHD-3D printed patches against *S. aureus* and *E. coli* bacteria. All scale bars indicate 10 mm.

	Sample	BC/PCL	BC/PCL+AMX	BC/PCL+AMP	BC/PCL+KAN
Bacterial Strain					
*S. aureus*		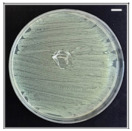	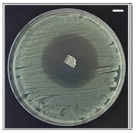	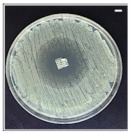	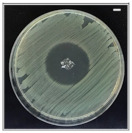
*E. coli*		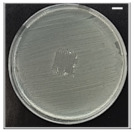	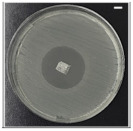	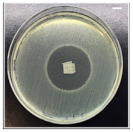	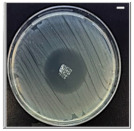

## Data Availability

The raw/processed data required to reproduce these findings cannot be shared at this time due to technical or time limitations. Data will be made available on request.
